# Supplementary material for: Reducing the risk of transfusion-transmitted infectious disease markers in blood and blood component donations: Movement from remunerated to voluntary, non-remunerated donations in Lithuania from 2013 to 2020
Source: PLoS One. 2022 Nov 15;17(11):e0277650. doi: 10.1371/journal.pone.0277650 (PMC9665384; doi:10.1371/journal.pone.0277650)
Supplement: S1 Table — (DOCX) [file pone.0277650.s001.docx]

**S1 Table. The number of donations with confirmed infectious disease markers according to the donation type in Lithuania from 2013 to 2020**

| **Year** | **Donations** | | **Confirmed markers** | | | | | **All donations** |
| --- | --- | --- | --- | --- | --- | --- | --- | --- |
|  |  |  | **HCV** | **HBV** | **HIV 1 and 2** | **Syphilis** | **Total** |  |
| 2013 | First time | Remunerated | 262 | 76 | 10 | 93 | 441 | 18480 |
|  |  | VNRD | 56 | 43 | 2 | 34 | 135 | 15143 |
|  | Repeat | Remunerated | 65 | 6 | 5 | 16 | 92 | 41008 |
|  |  | VNRD | 5 | 4 | 0 | 5 | 14 | 16338 |
|  | ***Total*** | | ***388*** | ***129*** | ***17*** | ***148*** | ***682*** | ***90969*** |
|  | | | | | | | | |
| 2014 | First time | Remunerated | 126 | 30 | 3 | 52 | 211 | 6314 |
|  |  | VNRD | 99 | 66 | 2 | 38 | 205 | 18754 |
|  | Repeat | Remunerated | 37 | 5 | 3 | 12 | 57 | 39415 |
|  |  | VNRD | 9 | 3 | 2 | 6 | 20 | 28897 |
|  | ***Total*** | | ***271*** | ***104*** | ***10*** | ***108*** | ***493*** | ***93380*** |
|  | | | | | | | | |
| 2015 | First time | Remunerated | 56 | 8 | 2 | 17 | 83 | 1940 |
|  |  | VNRD | 108 | 114 | 6 | 72 | 300 | 28053 |
|  | Repeat | Remunerated | 24 | 0 | 1 | 8 | 33 | 20695 |
|  |  | VNRD | 10 | 8 | 3 | 8 | 29 | 49824 |
|  | ***Total*** | | ***198*** | ***130*** | ***12*** | ***105*** | ***445*** | ***100512*** |
|  | | | | | | | | |
| 2016 | First time | Remunerated | 37 | 6 | 2 | 5 | 50 | 1125 |
|  |  | VNRD | 79 | 106 | 3 | 44 | 232 | 24618 |
|  | Repeat | Remunerated | 21 | 2 | 1 | 1 | 25 | 10953 |
|  |  | VNRD | 13 | 10 | 3 | 3 | 29 | 68479 |
|  | ***Total*** | | ***150*** | ***124*** | ***9*** | ***53*** | ***336*** | ***105175*** |
|  | | | | | | | | |
| 2017 | First time | Remunerated | 9 | 3 | 0 | 7 | 19 | 227 |
|  |  | VNRD | 108 | 87 | 1 | 44 | 240 | 21874 |
|  | Repeat | Remunerated | 5 | 0 | 0 | 0 | 5 | 3128 |
|  |  | VNRD | 17 | 9 | 6 | 7 | 39 | 74779 |
|  | ***Total*** | | ***139*** | ***99*** | ***7*** | ***58*** | ***303*** | ***100008*** |
|  | | | | | | | | |
| 2018 | First time | Remunerated | 0 | 0 | 0 | 0 | 0 | 1 |
|  |  | VNRD | 57 | 65 | 2 | 32 | 156 | 18970 |
|  | Repeat | Remunerated | 1 | 0 | 0 | 0 | 1 | 815 |
|  |  | VNRD | 14 | 6 | 6 | 16 | 42 | 82701 |
|  | ***Total*** | | ***72*** | ***71*** | ***8*** | ***48*** | ***199*** | ***102487*** |
|  | | | | | | | | |
| 2019 | First time | Remunerated | 0 | 0 | 0 | 0 | 0 | 2 |
|  |  | VNRD | 48 | 57 | 3 | 31 | 139 | 17509 |
|  | Repeat | Remunerated | 1 | 0 | 0 | 0 | 1 | 471 |
|  |  | VNRD | 7 | 4 | 4 | 9 | 24 | 89736 |
|  | ***Total*** | | ***56*** | ***61*** | ***7*** | ***40*** | ***164*** | ***107718*** |
|  | | | | | | | | |
| 2020 | First time | Remunerated | 0 | 0 | 0 | 0 | 0 | 0 |
|  |  | VNRD | 38 | 45 | 0 | 19 | 102 | 10812 |
|  | Repeat | Remunerated | 0 | 0 | 0 | 0 | 0 | 19 |
|  |  | VNRD | 6 | 5 | 4 | 4 | 19 | 85230 |
|  | ***Total*** | | ***44*** | ***50*** | ***4*** | ***23*** | ***121*** | ***96061*** |
|  | | | | | | | | |
