# Supplementary material for: Reducing the risk of transfusion-transmitted infectious disease markers in blood and blood component donations: Movement from remunerated to voluntary, non-remunerated donations in Lithuania from 2013 to 2020
Source: PLoS One. 2022 Nov 15;17(11):e0277650. doi: 10.1371/journal.pone.0277650 (PMC9665384; doi:10.1371/journal.pone.0277650)
Supplement: Attachment — Submitted filename: Response to Reviewer.docx [file pone.0277650.s002.docx]

Editors

PLOS ONE

May 9, 2022

Dear Editor,

We thank you and the reviewers for a thorough reading and constructive criticism of our manuscript and for the opportunity to revise and resubmit. We are pleased to submit the improved research article “Reducing the risk of transfusion-transmitted infectious disease markers in blood and blood component donations: movement from remunerated to voluntary, non-remunerated donations in Lithuania from 2013 to 2020” for your consideration for publication in PLOS ONE. On the following pages, you will find our response to the Editor comments.

On behalf of the authors, I thank you for your consideration of this resubmission. We appreciate your time and look forward to your response.

Sincerely,

Ass. Prof. Vytenis Kalibatas, MD, MPH, PhD (corresponding author)

Department of Health Management,

Lithuanian University of Health Sciences

Tilžės str. 18, LT-47181, Kaunas, Lithuania

E-mail: vytenis.kalibatas@lsmuni.lt

Journal Requirements

*1. When submitting your revision, we need you to address these additional requirements.*

*Please ensure that your manuscript meets PLOS ONE's style requirements, including those for file naming. The PLOS ONE style templates can be found at* 
[*https://journals.plos.org/plosone/s/file?id=wjVg/PLOSOne_formatting_sample_main_body.pdf*](https://journals.plos.org/plosone/s/file?id=wjVg/PLOSOne_formatting_sample_main_body.pdf)  *and* 
[*https://journals.plos.org/plosone/s/file?id=ba62/PLOSOne_formatting_sample_title_authors_affiliations.pdf*](https://journals.plos.org/plosone/s/file?id=ba62/PLOSOne_formatting_sample_title_authors_affiliations.pdf)

We thank you for the advices on the revision. We followed the templates and changed the figure file according to the requirements.

*2. In your Data Availability statement, you have not specified where the minimal data set underlying the results described in your manuscript can be found. PLOS defines a study's minimal data set as the underlying data used to reach the conclusions drawn in the manuscript and any additional data required to replicate the reported study findings in their entirety. All PLOS journals require that the minimal data set be made fully available. For more information about our data policy, please see* [*http://journals.plos.org/plosone/s/data-availability*](http://journals.plos.org/plosone/s/data-availability)*.*

*Upon re-submitting your revised manuscript, please upload your study’s minimal underlying data set as either Supporting Information files or to a stable, public repository and include the relevant URLs, DOIs, or accession numbers within your revised cover letter. For a list of acceptable repositories, please see* [*http://journals.plos.org/plosone/s/data-availability#loc-recommended-repositories*](http://journals.plos.org/plosone/s/data-availability#loc-recommended-repositories)*. Any potentially identifying patient information must be fully anonymized.*

We have uploaded our supplementary information in S1 Table. All relevant data are within the article and its supporting information.

3. Responses to Additional Editor Comments:

| **№** | **Comments** | **Responses** |
| --- | --- | --- |
| 1. | Serological test always carries an element of false positivity. Was any other test done to confirm the positive status of donor? | In the manuscript, we have used the word "confirmed", which we treated as "a priori" explanation that we provide the data only on confirmed TTI markers, not initially positive. Anyway, to make the text more understandable for all readers, we added some explanations regarding the confirmatory testing in the "Material and methods" section (125-134 lines in Manuscript; 125-133 lines in the Revised Manuscript with Track Changes). |
| 2. | All repetitions may be deleted. | Done.  We have removed all repetitions in the "Results" section and included the additional table in the Supporting Information. |
| 3. | The results sections has to be re-written and concise | Done.  We rewrote the "Results" section and concise the text. |
| 4. | Line 31, Please rewrite as HIV 1 and 2. | Done.  We corrected the marker in the “Abstract” section. |
| 5. | Line 207-215, If for statistical analysis, sample size is insufficient, need not look at the statistics. | Done.  We deleted sentences in lines 207-215, and added that due to insufficient sample sizes comparisons with VNRDs are not applicable (lines 179-180 in Manuscript; lines 208-209 in the Revised Manuscript with Track Changes). |
| 6. | Year wise statistics has not much relevance. It can be combined together. | We could agree with this comment if we emphasize a sufficient sample size of remunerated donations in some years. By combining some years (for example, 2013-2014; 2015-2016, etc.), we miss some important aspects of showing the breaking points from prevailing remunerated to VNRDs, and the shift towards the safest donation group - repeat VNRDs, which prevails starting from 2016. On the other hand, combining two or more years does not solve the problem of a sufficient sample size for first-time remunerated donations. In a manuscript, we provide the pooled data, combining all years, but we also think that data presented yearly give some useful information about donations' changes. |
| 7. | Line 205-208, where sufficient numbers not available, tests of significance carries no value. Such results may be omitted | Done.  We deleted sentences in lines 205-205, and added that due to insufficient sample sizes comparisons with VNRDs are not applicable (lines 179-180 in Manuscript; lines 208-209 in the Revised Manuscript with Track Changes). |
| 8. | Line 232- not complete | Done.  We added the year 2020 (line 209 in Manuscript; line 233 in the Revised Manuscript with Track Changes). |
| 9. | Were the TTI reactive donors contact and post test counselling done to assess the risk behaviors? | In answering the question, we provided an information in the “Discussion” section (lines 319-325 in Manuscript; lines 394 - 400 in the Revised Manuscript with Track Changes). |
| 10. | Line 297-98, what could be the reason for this finding? | Thank you for pointing it out. The sentence was incorrect: “repeat VNRDs are safer than repeat non-remunerated donations”. We corrected the sentence (lines 272-273 in Manuscript; lines 346 - 347 in the Revised Manuscript with Track Changes). |
| 11. | Help of a statistician may be sought for better presentation of the results. | We consulted with a statistician and made some changes in calculations (z-test instead of chi-square test for comparisons of prevalence), as well as in the interpretation of the results. |
